# Impact of paediatric antimicrobial stewardship program in haematogenous bone and joint infections

**DOI:** 10.1007/s00431-025-06258-7

**Published:** 2025-06-18

**Authors:** Aina Font, Marta Agüera, María Ríos-Barnés, Anna Gamell, David Moreno-Romo, Maria Goretti López-Ramos, Manuel Monsonís, Victoria Fumadó, Silvia Simó-Nebot, Cesar G. Fontecha, Clàudia Fortuny, Daniel Echeverría-Esnal, Antoni Noguera-Julian, Eneritz Velasco-Arnaiz

**Affiliations:** 1 Pharmacy Department, Consorci Hospitalari de Vic, Vic, Spain; 2 Present Address: Medicine and Translational Research, Barcelona University, Barcelona, Spain; 3 Pediatrics Department, Hospital Sant Joan de Déu, Pg. de Sant Joan de Déu, 2, 08950 Esplugues de Llobregat, Barcelona, Spain; 4 Infectious Diseases Department, Hospital Sant Joan de Déu and Institut de Recerca Sant Joan de Déu, Barcelona, Spain; 5 Pediatric Orthopaedic Surgery and Traumatology Department, Hospital Sant Joan de Déu, Barcelona, Spain; 6 Pharmacy Department, Hospital Sant Joan de Déu, Barcelona, Spain; 7 Microbiology Department, Hospital Sant Joan de Déu, Barcelona, Spain; 8 Centre for Biomedical Network Research On Epidemiology and Public Health (CIBERESP), Madrid, Spain; 9 Department of Surgery and Medical and Surgical Specialities, Faculty of Medicine and Health Sciences, University of Barcelona, Barcelona, Spain; 10 Pharmacy Department, Hospital del Mar, Barcelona, Spain; 11 Infectious Pathology and Antimicrobials Research Group (IPAR), Universitat Pompeu Fabra, Barcelona, Spain

**Keywords:** Infectious diseases, Paediatric care, Antibiotics, Bone and joint infections

## Abstract

Antimicrobial stewardship programs (ASP) reduce the inappropriate use of antimicrobial agents. Traditionally, paediatric osteoarticular infections have been treated with prolonged intravenous antibiotics; however, evidence suggests that shorter intravenous therapy followed by oral narrow-spectrum antibiotics is effective. The aim of this study is to assess the impact of an ASP on the treatment of acute haematogenous bone and joint infections (AH-BJI) in children.We performed a single-centre quasi-experimental study comparing antibiotic use in paediatric [0–18 years] inpatients with AH-BJI before (2015–2016 period 1 [P1]) and after (2017-june 2023 period 2 [P2]) the implementation of a multifaceted hospital ASP with post-prescription review with feedback as the primary strategy. ASP also promoted a first-line empiric antibiotics change in the local protocol in June 2020. The study describes and compares the demographic and clinical characteristics among patients who were recruited prospectively during their hospital admission or in outpatient clinics. The primary outcomes were the length of antibiotic therapy, length of hospital stay, sequelae, readmission and fatality rates. Two-hundred-eighty-five patients were included (60 in P1 and 225 in P2). The length of parenteral antibiotic treatment and the length of hospital stay were significantly lower in P2 (median [IQR] days, P1: 8.5[7.0–12.0] vs P2: 7[4.5–8.0], *p* < 0.001; and P1: 8.5[7.0–11.0] vs P2: 7[5.0–9.0], *p* < 0.001, respectively). After June 2020, 3rd generation cephalosporin use decreased in patients < 5 years old (96/119[81%] vs 6/57[10%] cases; *p* < 0.001). The rates of sequelae, readmission (2/60[3.3%] in P1 and 8/225[3.6%] in P2) and mortality remained unchanged. *Conclusion*: After ASP implementation, the length of parenteral antibiotic treatment, length of hospital stay, and 3rd generation cephalosporin use in children with AH-BJI were reduced safely.

**Table:** 

**What is Known:** • *Antimicrobial Stewardship Programmes (ASPs) have been shown to be effective for optimising antibiotic prescriptions and reducing antimicrobial resistance. There is still limited literature about ASPs in the paediatric population.* • *Paediatric bone and joint infections were traditionally treated with prolonged intravenous antibiotics. However, current evidence suggests that shorter intravenous therapy followed by oral narrow-spectrum antibiotics is equally effective.*
**What is New:** • *Implementing an ASP with post-prescription review with feedback as main strategy, in a tertiary paediatric hospital, safely reduced the duration of intravenous treatment and length of hospital stay for children with acute haematogenous bone and joint infections (AH-BJI).* • *The ASP helped to reduce the use of 3rd generation cephalosporin in children with AH-BJI.*

**Supplementary Information:**

The online version contains supplementary material available at 10.1007/s00431-025-06258-7.

## Introduction

Antimicrobial stewardship programmes (ASPs) have emerged as a crucial strategy to reduce the inappropriate use of antimicrobial agents [1]. The evidence supporting their effectiveness in paediatrics is growing [2].

Acute haematogenous bone and joint infections (AH-BJI) have traditionally been treated with parenteral antibiotics for several weeks or months, representing a potential target for paediatric ASP [3, 4]. Recent studies and guidelines recommend intravenous (IV) therapy for a few days followed by oral therapy with first generation cephalosporins, anti-staphylococcal penicillin or clindamycin in most children with uncomplicated AH-BJI. This approach is based on the aetiology of these infections and the favourable outcomes with early oral switch and shorter antibiotic durations [5–15]. Total 2-to 3-week courses for septic arthritis (SA) and 3–4 weeks for osteomyelitis (OM) are the current standard of care, if there are no complications, in which case longer regimens are still recommended [5–7, 12, 13]. A clinical trial involving adults showed that oral antibiotic treatment was as effective as intravenous therapy for complex bone and joint infections, resulting in fewer complications and shorter hospital stays. A recent clinical trial in a paediatric population assessed the use of exclusive oral treatment with favourable results [16–18].

Antibiotic selection and dosing, including in patients with penicillin allergy labels, along with intravenous-to-oral transitions, exclusive oral treatment, treatment duration and therapy monitoring have all been identified as potential areas for improvement in the management of AH-BJI in paediatric patients [18–22]. Clinical pathways and diagnostic algorithms, guideline implementation [19, 23], prospective audit and feedback [24] and utilization of methicillin-resistant *Staphylococcus aureus* (MRSA) nasal swab to guide de-escalation of anti-MRSA antibiotics are some of the ASP interventions reported [25].

We aimed to evaluate the impact of a post-prescription audit with feedback (PPRF)-based multifaceted hospital ASP on the treatment of paediatric AH-BJI concerning antibiotic use profile, antibiotic treatment duration and length of hospital stay.

## Materials and methods

### Study design

This was a single-centre, quasi-experimental study comparing antibiotic use in paediatric inpatients with AH-BJI before and after implementing a multifaceted hospital ASP in 2017. The pre-intervention period (P1) encompasses January 2015 to December 2016, while the post-intervention (P2) spans January 2017 to June 2023.

### Setting and population

The study included paediatric patients from birth up to 18 years of age, diagnosed with AH-BJI (OM, SA, osteoarthritis [OA], or spondylodiscitis [SD]) and admitted to either the general paediatric ward or the orthopaedics and traumatology ward at Sant Joan de Déu Hospital, a tertiary-care maternal and children’s hospital in Barcelona, Spain. Patients were recruited prospectively during their hospital admission or in outpatient clinics. All patients diagnosed with AH-BJI were included, regardless of their immunisation or immunological status. AH-BJI diagnosis was made following the national consensus [26]. Acute osteoarticular infection was classified as a process with less than 14 days of symptomatology and community-acquired [26]. SA and OM were defined as the presence of compatible symptoms (fever, pain, joint swelling, impaired function) and radiological findings (ultrasound, MRI or gammagraphy), with or without a microbiological isolation and favourable progression following antibiotic therapy. Exclusion criteria were diagnosis of non-acute BJI (more than 14 days from the onset of symptoms), orthopaedic implant-associated infections or other non-infectious conditions (e.g. chronic recurrent multifocal osteomyelitis). Patients were followed for at least three months post-discharge.

### Intervention

The hospital ASP (Programa de Optimización del Uso de Antimicrobianos Sant Joan de Déu, PROA-SJD) was first implemented in January 2017 by a multidisciplinary team, using PPRF as the primary strategy, as previously reported [27, 28], and in line with national recommendations [29]. Before ASP implementation, the hospital infectious diseases department provided recommendations for AH-BJI patient management upon physician request. Since January 2017, all AH-BJI patients have been reviewed daily by a paediatric infectious disease specialist from the ASP team. Prescriptions are evaluated as ‘optimal’ or’non-optimal’ based on a set of criteria, and the complete evaluation is included in an explicitly designed form for the ASP in the patient’s clinical chart [28, 30]. Non-optimal prescriptions receive recommendations for optimization written in the electronic medical record and during clinical rounds, with acceptance at the prescriber’s discretion. In addition, a paediatric traumatology specialist has been appointed as referral for AH-BJI to facilitate regular communication between the ASP team and the traumatology-orthopaedics department. Weekly case discussions and quarterly meetings to identify areas for improvement and to reinforce the collaboration and engagement with the ASP team are held. An example of this is the creation of specific pre-established laboratory orders in the electronic medical program. These requests include the necessary biochemical and microbiological tests for each type of sample. Additionally, pre-set protocols determining the most appropriate empirical antibiotic and calculating the correct dosages based on patient age and weight are featured in the e-prescribing program, facilitating accurate prescription and dosing. The importance of obtaining blood cultures before starting antibiotic therapy was emphasised to all clinical teams.

In addition, in June 2020, five years after the previous version, the local guidelines for AH-BJI treatment were revised. The combination of a 3rd generation cephalosporin and cloxacillin as empirical first-line antibiotic treatment was replaced by cefuroxime in patients between 3 months and 5 years of age, and cloxacillin was replaced by cefazolin in patients older than 5 (supplementary Table 1). The recommendations for switching from IV to oral therapy as well as the overall duration of the antibiotic treatment remained unchanged. In uncomplicated cases, oral switch is to be considered 24 to 48 h after the resolution of fever with maintained improvement in pain, mobility and swelling for at least 24 h, as well as a significant decrease (e.g.30%) in C reactive protein [6]. The recommended total duration of IV plus oral antibiotic therapy is 3–4 weeks for OM, 2–3 weeks for SA, 4–6 weeks for hip/shoulder OA, 6 weeks for SD, and at least 4–6 weeks in complicated AH-BJI. The patient’follow-up includes appointments with the infectious diseases and traumatology-orthopaedics teams.

### Study variables and outcomes

Demographic, clinical, microbiological and treatment data were obtained from electronic medical chart records. Microbiological data included the diagnostic tests performed and the microorganisms identified by culture or by nucleic-acid amplification tests (polymerase chain reaction-PCR). Regarding the treatment, the antimicrobial agents that were used, the length of parenteral antibiotic treatment (LPA), the length of oral antibiotic treatment (LOA), the total length of treatment (LOT) and the length of hospital stay (LOS) were collected. LPA, LOA and LOT were defined as the number of consecutive days a patient received systemic antibiotics, irrespective of the number of agents or dose, by IV or oral route, or by the sum of both, respectively. LOS was defined as the length of an inpatient episode of care, calculated from admission to discharge day, based on the number of nights spent in the hospital [31]. Complications were considered to include: pyomyositis, soft tissue abscesses and subperiosteal abscesses and septic embolus pneumonia. Sequelae during follow-up, readmission rates (RR), and fatality rates (FR) were considered as balancing measures, assuring that ASP interventions did not lead to increased harm. RR was calculated as the percentage of non-scheduled readmissions related to AH-BJI within 90 days after discharge, and FR was defined as the percentage of all-cause deaths within 365 days post-discharge.

### Statistical analyse

Statistical analyses were performed using SPSSv29.0.2 (IBM Corporation, Armonk, NY, USA). Categorical variables were presented as proportions with a 95% confidence interval (95% CI) and compared using Chi-squared and Fisher tests. Quantitative variables were reported as median with interquartile range (IQR) and compared using U-Mann–Whitney or Kruskal–Wallis tests. We compared LPA, LOA, LOT and LOS in P1 and P2. Antibiotic profile use was compared before (01/15–06/20) and after (07/20–12/23) local protocol update. The empirical treatment recommendations of ASP were applied to all patients; however, adherence to these recommendations was at the discretion of the treating clinicians and was not formally evaluated. A p-value < 0.05 was considered statistically significant.

### Ethics Statements

This study was approved by the Sant Joan de Déu Research Foundation Ethics Committee [EOM-16–23]. The research followed the Declaration of Helsinki and national and institutional standards. Informed consent was obtained from parents/guardians, and informed assent from patients > 11 years at admission.

## Results

During the study period, 285 patients were admitted with a diagnosis of AH-BJI: 60 in P1 and 225 in P2 (supplementary Fig. 1). Table 1 shows the main demographic and clinical characteristics of the study participants. The most common diagnosis in both periods was OM (133/285, 46.7%), followed by SA (112/285, 39.3%), SD (21/285, 7.4%) and OA (19/285, 6.7%). In P2, the proportion of cases of OM was higher than in P1, but the difference was not statistically significant (p = 0.08). The main sites of OM were the lower limbs (81/133, 60.9%) and particularly large bones: femur and tibia (50/133, 37.6%). In patients with SA, the hip was the most affected joint (50/112, 44.6%). Supplementary Table 1 shows the comparison of the distribution of localizations by diagnosis between P1 and P2.

**Table 1:** Demographic and clinical characteristics of the patients with acute haematogenous bone and joint infections during period 1 (2015–2016) and period 2 (2017- June 2023). Data are shown as number (percentage) or median (interquartile range)

	Period 1 *n* = 60	Period 2 *n* = 225	*p*-value
Age (years)	1.9 (1.2–6.5)	2.9 (1.3- 10.6)	0.12
< 5 years old	42 (70.0%)	132 (58.7%)	0.19
Sex (male)	30 (50.0%)	152 (67.5%)	0.01
*Clinical characteristics*			
Fever	35 (58.3%)	147 (65.3%)	0.39
Limp	41 (68.3%)	152 (67.5%)	0.68
Swelling	25 (41.7%)	96 (42.7%)	0.87
Immobility	55 (91.7%)	189 (84.0%)	0.13
Mobility limitation	32 (53.3%)	127 (56.4%)	0.68
Refusal to sit	14 (23.3%)	16 (7.1%)	< 0.001
*Analytical parameters*			
ESR (mm/h) (P1 n = 39; P2 *n* = 137)	28.0 (19.0–37.0)	22.0 (10.0–34.0)	0.08
CRP (mg/L) (P1 n = 60; P2 *n* = 141)	30.7 (10.6–74.0)	48.0 (20.8–99.0)	0.03
Procalcitonin (ng/mL) (P1 n = 24; P2 n = 65)	0.10 (0.05–0.25)	0.09 (0.05–0.97)	0.91
*Diagnosis*			
Septic arthritis	24 (40.0%)	88 (39.2%)	0.002
Osteomyelitis	22 (36.7%)	111 (49.3%)	
Osteoarthritis	3 (5.0%)	16 (7.1%)	
spondylodiscitis	11 (18.3%)	10 (4.4%)	
*Complications*			
Pyomyositis	0 (0.0%)	9 (4.0%)	0.11
Soft tissue abscess	1 (1.7%)	22 (9.8%)	0.04
Subperiosteal abscess	5 (8.3%)	24 (10.7%)*	0.59
Septic embolus pneumonia	0 (0.0%)	1 (0.4%)	0.60
Others **	5 (8.3%)	7 (3.1%)	0.07
PICU admission	2 (3.3%)	2 (0.9%)	0.15
Sequelae after antibiotic ^	3 (5.0%)	15 (6.7%)	0.64

*ESR* erythrocyte sedimentation rate, *CRP* C reactive protein, *MSSA* methicillin-susceptible *Staphylococcus aureus*, *MRSA* methicillin-resistant *Staphylococcus aureus.* *One patient had two complications: soft tissue abscess and subperiosteal abscess. **Others: sepsis, intramedullary abscess, intraosseous abscess, pathologic fracture, joint effusion and hematoma, hip subluxation. ^Sequelae after 3 months of follow-up: dysmetria, limitation of mobility, pain, lameness

Seventy-four patients (25.9%) experienced complications related to AH-BJI during their admission. Only soft tissue abscesses were significantly more frequent in the second study period (Table 1). All 6 patients with soft tissue abscesses and subperiosteal abscesses in P1 were treated conservatively, while 14 out of 46 (30%) in P2 required surgery. No sequelae were observed in either period after completion of the antibiotic treatment.

Most patients underwent one or more diagnostic procedures (239/285 [83.9%] blood cultures, 132/285 [46.3%] joint puncture and 36/285 [12.6%] bone puncture). The diagnostic and microbiological characteristics of the study population are reported in Table 2. A bacterial pathogen was identified in 151/285 (53.0%) cases. The most common etiological agent was MSSA (65/285, 22.8%), followed by *Kingella kingae* (31/285, 10.9%), exclusively found in children under four years of age. Blood cultures were positive in 70 out of 239 (29.3%) cases, 47/70 (67.1%) with *S*. *aureus* isolation; focus sample cultures yielded positive results in 73 out of the 149 cultured (49.0%)*. Kingella* was detected by PCR in synovial fluid or bone in most cases and in only 1/31 (3.2%) in blood culture. We only included the microbiological diagnostic procedures, because we considered them to be the most relevant to the issue under the study. However, X-rays, ultrasounds, MRIs or gamagraphies were performed during the diagnostic and therapeutic processes.

**Table 2:** Diagnostic and microbiological data of patients with acute haematogenous bone and joint infections in period 1 (2015-2016) and period 2 (2017- June 2023). Data shown as number (percentage)

	Period 1 *n*= 60	Period 2 *n*= 225	*p*-value
*Diagnostic procedures*			
Blood culture	43 (71.7%)	196 (87.1%)	0.004
Arthrocentesis/arthrotomy	30 (50.0%)	102 (45.3%)	0.62
Bone puncture	5 (8.3%)	31 (13.8%)	0.36
*Pathogen identification*			
Blood culture (n=239)	12/43 (27.9%)	58/196 (29.6%)	0.83
Synovial fluid culture (n= 113)	11/25 (44.0%)	38/88 (43.2%)	0.94
Bone sample culture (n=36)	5/5 (100%)	19/31 (61.3%)	0.15
Synovial fluid PCR (n= 93) **	10/22 (45.4%)	34/71 (47.9%)	0.84
*Etiological agent*			
Unknown	28 (46.7%)	106 (47.1%)	0.66
MSSA	12 (20.0%)	53 (23.6%)	0.56
MRSA	2 (3.3%)	9 (4.0%)	
* Kingella kingae*	9 (15%)	22 (9.8%)	
* S.pneumoniae*	4 (6.6%)	7 (3.1%)	
* S.pyogenes*	1 (1.7%)	10 (4.4%)	
Other*	4 (6.7%)	18 (8.0%)	

*MSSA* methicillin-susceptible *Staphylococcus aureus,* *MRSA* methicillin-resistant *Staphylococcus aureus*. *Other: *Streptococcus agalactiae, Salmonella*, other enterobacteria. ** *MSSA* *n*=12; *Kingella kingae n*=24, *S. pneumoniae n*=8

Regarding empirical antibiotic use, the percentage of patients aged up to 5 years old with 3rd generation cephalosporin coverage decreased significantly from 81.0% to 10.0% (*p* < 0.001) after 2020. In patients older than 5 years of age, cloxacillin use decreased by 60.0% (*p* < 0.001), whereas cefazolin’s use increased by 58.0% (*p* < 0.001) over the same period.

The duration of the antibiotic treatment (LPA, LOA and LOT) and the LOS in the whole study cohort and according to the final diagnosis are shown in Table 3. Overall, the median LPA and the median LOS were significantly shorter in P2 compared to P1, and in both SA and OM. LOT remained similar while a statistically significant difference was found in comparison of the LOA between P1 and P2.

**Table 3:** Comparison of length of antibiotic treatment (parenteral, oral and total) and hospital stay between period 1 (2015–2016) and period 2 (2017–June 2023) in the whole study population, and according to the final diagnosis. Data shown as median (interquartile range) days

	P1 *n* = 60	P2 *n* = 225	*p*-value
LPA	9 (7–12)	7 (5–8)	< 0.001
LOA	21 (15–2)	21 (15–35)	0.03
LOT	27 (23–34)	28 (21–42)	0.46
LOS	9 (7–11)	7 (5–9)	< 0.001
Diagnosis			
*Septic arthritis*	*n* = 24	*n* = 88	
LPA	8 (7–9)	5 (4–8)	< 0.001
LOA	15 (14–21)	15 (14–21)	0.99
LOT	24 (21–29)	21 (16–31)	0.19
LOS	8 (7–9)	6 (5-9)	< 0.001
*Osteomyelitis*	*n* = 22	*n* = 111	
LPA	9 (6–12)	7 (5–8)	0.002
LOA	21 (20–23)	25 (21–37)	0.02
LOT	28 (25–37)	31 (25–45)	0.34
LOS	9 (7–12)	7 (5–9)	0.02
*Osteoarthritis*	*n=3*	*n = 16*	
LPA	15 (7–18)	7 (5–9)	0.64
LOA	21 (15–29.5)	32 (17–42)	0.42
LOT	30 (28–44.5)	40.5 (29–49)	0.79
LOS	18 (7–21)	7 (5–10)	0.08
*Spondylodiscitis*	*n* = 11	*n* = 10	
LPA	10 (7–14)	7 (6–9)	0.10
LOA	17 (16–49)	63 (51–84)	0.005
LOT	27 (21–49)	70 (57–92)	0.002
LOS	10 (7–15)	7 (7–8)	0.05

*LPA*, length of parenteral antibiotic treatment; *LOA*, length of oral antibiotic treatment; *LOT*, total length of antibiotic treatment; *LOS*, length of hospital stay

Under further analysis, we found that patients with a complicated course with soft-tissue or subperiosteal abscesses —comprising 52 participants, 46 of whom were from P2— accounted for the longest LOA and LOT (median [IQR] LOA 41 [26–51] days; median [IQR] LOT 47 [33–63] days, respectively). In the subgroup of patients with documented bacteraemia, the median LPA in P2 was also shorter than in P1, but the 2-day reduction in the median LOS did not reach statistical significance (Table 4).

**Table 4:** Comparison of the length of antibiotic treatment and hospital stay in patients with positive blood culture between period 1 (2015–2016) and period 2 (2017–June 2023). Data shown as median (interquartile range)

	P1 *n* = 12	P2 *n* = 58	*p*-value
LPA (days)	11 (8–17)	9 (7–11)	0.04
LOA (days)	21 (15–39)	28 (21–42)	0.34
LOT (days)	33 (27–54)	35 (28–51)	0.94
LOS (days)	11 (7–17)	9 (7–12)	0.11

*LPA*, length of parenteral antibiotic treatment; *LOA*, length of oral antibiotic treatment; *LAT*, total length of treatment; *LOS*, length of hospital stay

At the end of follow-up, 18 out of the 285 patients (6.3%) exhibited sequelae, with no differences between P1 and P2 (Table 1). Mortality was zero in both study periods and the readmission rate remained unchanged (2/60 [3.3%] in P1 and 8/225 [3.6%] in P2, respectively).

## Discussion

The literature on the efficacy of ASP in children and adolescents with BJI remains limited [20, 32]. Implementing evidence-based treatment guidelines with a multidisciplinary team may contribute to a more efficient diagnosis of AH-BJI, an increased rate of causative organism identification, better adherence to initial antibiotic recommendations, and fewer antibiotic changes during treatment [32]. Our ASP employed educational, consensus-forming, and persuasive strategies, and promoted interaction and feedback between an infectious disease physician and the clinicians involved in the diagnosis, treatment, and follow-up of children with AH-BJI. The results of the study confirm that a multifaceted PPRF-based hospital ASP can successfully reduce LPA and LOS in paediatric inpatients with AH-BJI. Moreover, it can facilitate prompt compliance with guideline updates, as shown by the shift in antibiotic use profile after 2020. Our cohort shares the main epidemiological and clinical characteristics with previous paediatric studies [7, 20, 33]; therefore, we believe that the results could be applicable to high-income environments with low rates of MRSA infections.

Determining the causative microbiological agent contributes to ASP interventions, by guiding targeted antibiotic selection, as well as establishing the appropriate route and duration of treatment. In line with contemporary diagnostic strategies that promote a less invasive approach [13, 34], obtaining bone samples in uncomplicated OM cases and in patients with SD who had no clinical indication for surgery was unusual. However, arthrocentesis and bone biopsy were performed in 15% and 9% more cases, respectively, compared to a recent Danish trial on the efficacy and safety of initial oral antibiotics in uncomplicated BJI, possibly due to the inclusion of patients with more severe clinical presentation in our study [25]. On the other hand, an increasing tendency to order blood cultures at admission was observed, a recommendation reinforced by the PROA-SJD. Blood cultures with a sufficient volume are crucial [35] particularly when a non-invasive approach is chosen and samples from the infected bone or joint tissue are not available, given that bacteraemia is the main pathogenic mechanism of BJI in children [36, 37]. Ultimately, the microbiological aetiology was identified in more than 50% of the patients in both study periods, a considerably higher proportion—up to double—than that reported in other studies [7, 25, 38]. Overall, nearly a quarter of the cases were caused by *S. aureus* (half of the microbiologically confirmed cases) and only 14.5% of these isolates were methicillin resistant. *K. kingae* was the second most common agent and being a difficult-to-grow microorganism [39, 40], it was identified almost universally by PCR in focus samples, and exclusively in patients younger than four years of age, as reported in the literature [7, 20, 33, 39, 41]. This observation underscores the usefulness of including molecular techniques in the diagnostic algorithms of AH-BJI, as it is recognized that infections caused by *K. kingae* usually exhibit less aggressive behaviour and can be treated with short parenteral courses or, indeed, directly with oral antibiotics [10, 11, 42].

In uncomplicated AH-BJI cases showing clinical improvement, oral treatment has been shown to be clinically equivalent to prolonged IV therapy, safe and associated with fewer complications, shorter admission duration, and lower costs [6, 14, 15, 43, 44]. However, many of the studies supporting the early switch to oral antibiotics were conducted in relatively homogeneous populations, in terms of both the infection aetiology and the non-severe clinical presentation, or under clinical trial conditions [25]. Our study contributes to the existing knowledge by providing real-world experience of the feasibility and benefits of this strategy in a large paediatric cohort, with a significant reduction of the hospital stay and, potentially, associated complications. The reduction of the LPA following the development of the ASP led to earlier hospital discharge and subsequent decrease in LOS, without extending the total duration of the antibiotic treatment. In fact, the median LOT before ASP implementation was already within the currently recommended durations. We hypothesize the putative impact of on-demand support from the infectious diseases team, alongside the publication, shortly before the first study period, of a national consensus document on the treatment of uncomplicated OM and AS with contributing authorship by traumatologists and a paediatrician from our hospital [6].

The rate of bacteraemia in our cohort is consistent with a previous national series, in which 24% patients had a positive blood culture [7], and contrasts with the 17% and 15% rates of the ELECTRIC study [21] and of the oral versus IV empirical antibiotics trial, respectively [25]. The reduction of LPA and LOS in our study was also observed in patients with a positive blood-culture. Bacteraemia may be a surrogate marker of the severity of clinical presentation; however, it is not, on its own, a criterion to rule out early transition to oral therapy, [42, 43] or, according to Nielsen’s trial, to preclude exclusively oral therapy [25].

The reduced LPA after ASP implementation might have resulted in fewer catheter-days and, therefore, in fewer days at risk of IV line-related complications [15]. Moreover, after the local guideline update in 2020, cephalosporins and cloxacillin was reduced drastically in favour of narrower spectrum cephalosporins, like other studies conducted in smaller cohorts [21]. Due to the dosage of cefuroxime and cefazolin, the number of daily IV administrations also decreased from 6 to 3 in children from 3 months to 5 years old, and from 4 to 3 in those older than 5. Minimizing the number of IV antibiotic doses is particularly relevant in the youngest children to reduce IV line-related complications.

In recent years, both an increase in the incidence of OM and a more accurate detection of complications, such as soft tissue abscesses, have been described [45, 46]. It has been suggested that this may be due to advances in imaging and microbiological techniques, which have led to a higher diagnostic yield [47, 48]. Unfortunately, we did not have information about the rate of Panton-Valentine leukocidin production in S. aureus AH-BJI or other virulence factors that contribute to a greater severity and a more complicated course and, consequently, to the need for longer antibiotic durations [49].

Up to 10% of children and adolescents with AH-BJI experience clinical and radiological sequelae, including dysmetria, reduced mobility, and chronic osteomyelitis [42, 50, 51]. The rate of sequelae in our cohort remained stable and within the expected range according to contemporary data during both periods. Following the study, we promoted the creation of a specific outpatient’clinic for the follow-up of children with AH-BJI after hospital discharge. The clinic is run collaboratively by a paediatric infectious diseases’specialist and a paediatric traumatologist, both of whom form a stable, integrated team. We believe that this intervention can improve patient care and help reduce variability in post-hospitalization antibiotic treatment management.

Our study has several limitations, namely the limited sample size, unbalanced between period 1 and 2 as per real-life implementation of the ASP, and its single-centre design, which may hinder the applicability of its findings to other settings. Including patients from birth in our study could cause confusion due to their different characteristics, such as longer IV therapy or different empirical antibiotic regimen. However, our aim was to assess an ASP in a real-life setting where patients of all ages are admitted to our hospital. Indeed, despite of including patients aged up to 3 months we observed a reduction in the length of parenteral antibiotic therapy and hospital length of stay, which we believe is more significant. Treatment recommendations were based on our local microbial epidemiology and, consequently, may not be appropriate for other areas. Due to information system constraints, we could not calculate the days-of-treatment (DOT) per 1000 hospital stays. Instead, LOT was used as a representative measure of the ASP’s impact. A formal cost–benefit analysis was not conducted, so we could not verify a reduction in healthcare costs following ASP implementation. Given the use of low-cost medications, any impact on overall pharmaceutical expenses was likely to be minimal. However, it would be of interest to study the potential reduction in indirect costs, especially LOS. The impact of the SARS-CoV-2 pandemic was not specifically addressed, nor were possible advances in diagnostic and surgical techniques and staff turnover [52]. Finally, we did not measure prescribers’ satisfaction or the level of acceptance of ASP recommendations. The adherence to these recommendations was not systematically monitored due to constraints in time and resources. Furthermore, the decision to follow them was left to the discretion of the treating clinicians. However, given the favourable outcomes observed, it is reasonable to assume that adherence and acceptance were satisfactory.

In conclusion, a PPRF-based multifaceted ASP incorporating a bundle of interventions addressed to traumatologists and paediatricians, effectively and safely reduced the LPA, the LOS and the use of 3rd generation cephalosporin in a large cohort of children with AH-BJI at a referral children’s hospital over a 5-year period following the program’s implementation. Further studies are needed to identify the best ASP interventions and to ensure and sustain their success over time.

## Supplementary Information

Below is the link to the electronic supplementary material.

Supplementary file 1 (DOCX 64.5 KB)

## Data Availability

No datasets were generated or analysed during the current study.

## Abbreviations

AH-BJI: Acute haematogenous bone and joint infections
ASP: Antimicrobial stewardship programme
DOT: Days-of-treatment
ESR: Erythrocyte sedimentation rate
FR: Fatality rate
IQR: Interquartile range
IV: Intravenous
LOS: Length of hospital stay
LOA: Length of oral antibiotic treatment
LOT: Length of total antibiotic treatment
LPA: Length of parenteral antibiotic treatment
MSSA: Methicillin-susceptible *Staphylococcus aureus*
MRSA: Methicillin-resistant *Staphylococcus aureus*
OA: Osteoarthritis
OM: Osteomyelitis
PPRF: Post-prescription review with feedback
PROA-SJD: Programa de Optimización del Uso de Antimicrobianos Sant Joan de Déu
PCR: Polymerase chain reaction
RR: Readmission rate
SA: Septic arthritis
SD: Spondylodiscitis
