# Supplementary material for: Impact of paediatric antimicrobial stewardship program in haematogenous bone and joint infections
Source: Eur J Pediatr. 2025 Jun 18;184(7):426. doi: 10.1007/s00431-025-06258-7 (PMC12174213; doi:10.1007/s00431-025-06258-7)
Supplement: Supplementary file 1 — (DOCX 64.5 KB) [file 431_2025_6258_MOESM1_ESM.docx]

**Supplementary table 1.** Empiric antibiotic therapy regimen before and after change of protocol in 2020.

|  | **2015-2019** | | **2020-2023** | |
| --- | --- | --- | --- | --- |
| ***IV empiric antibiotic therapy*** | **<1 month** | 3^rd^ generation cephalosporin (cefotaxime) + cloxacillin  Alternative: clindamycin + gentamycin | **<1 month** | 3^rd^ generation cephalosporin (cefotaxime) + cloxacillin  Alternative: clindamycin + gentamycin |
|  |  |  | **1 –3 months** | 3^rd^ generation cephalosporin (ceftriaxone) + cloxacillin  Alternative: clindamycin + gentamycin |
|  | **1 month - 5 years old** | 3^rd^ generation cephalosporin (ceftriaxone) + cloxacillin  Alternative: amoxicillin-clavulanic or cefuroxime | **3 months – 5 years old** | 2^nd^ generation cephalosporin (cefuroxime)  Alternative: ciprofloxacin or co-trimoxazole |
|  | **≥ 5 years old** | Cloxacillin  Alternative: cefazolin | **≥ 5 years old** | 1^st^ generation cephalosporin (cefazolin)  Alternative: vancomycin or clindamycin |
| ***Oral empiric antibiotic therapy*** | **< 5 years old** | Amoxicillin-clavulanic | **<2 years old** | Amoxicillin-clavulanic |
|  | **≥ 5 years old** | Cefadroxil | **≥ 2 years old** | Cefadroxil |

**Supplementary Figure 1.** Flow Diagram of Patient Selection


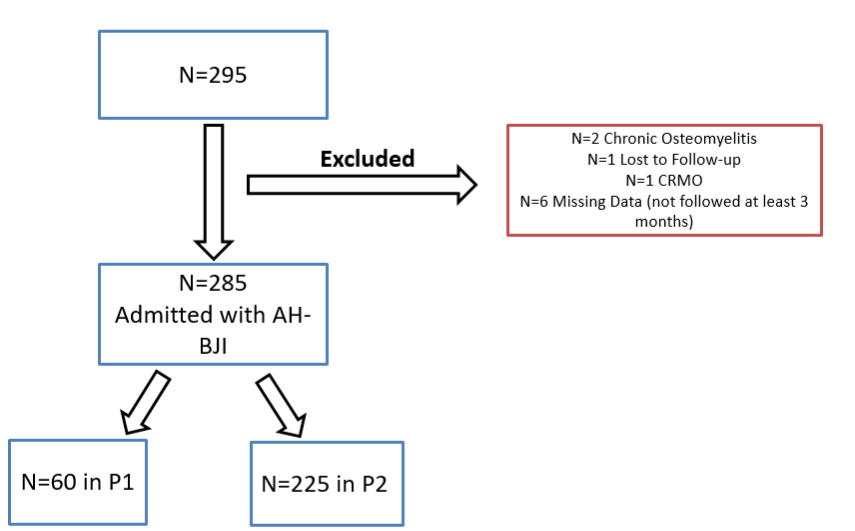


*AH-BJI: acute hematogenous bone and joint infections; CRMO: Chronic Recurrent Multifocal Osteomyelitis; P1: period 1 [2015-2016]; P2: period 2 [2017-june 2023].

**Supplementary Table 2.** Comparison of the locations of the AH-BJI in period 1 (2015-2016) and period 2 (2017- June 2023).

|  | **Period 1**  **n=60** | **Period 2**  **n=224 **** | **p-value** |
| --- | --- | --- | --- |
| ***Location of SA/OA*** | ***n=27*** | ***n=103*** |  |
| **Shoulder** | 4 (14.8%) | 7 (6.8%) | p>0.20*** |
| **Elbow** | 0 (0.0%) | 7 (6.8%) | 0.05< p<0.10*** |
| **Wrist** | 0 (0.0%) | 2 (1.9%) | 0.15< p<0.20*** |
| **Hip** | 12 (44.5%) | 38 (36.9%) | 0.47^ |
| **Knee** | 6 (22.2%) | 37 (35.9%) | 0.18^ |
| **Ankle** | 5 (18.5%) | 10 (9.8%) | 0.20<p<0.25*** |
| **Other** | 0 (0.0%) | 2 (1.9%) | 0.15< p<0.20*** |
| ***Location of SD*** | ***n=11*** | ***n=10*** |  |
| **Lumbar** | 10 (90.9%) | 10 (100%) | 0.10< p<0.15*** |
| **Dorsal** | 1 (9.1%) | 0 | 0.10< p<0.15*** |
| ***Location of OM*** | ***n=22*** | ***n=111*** |  |
| **Femur** | 1 (4.5%) | 25 (22.5%) | 0.07 ⸸ |
| **Tibia** | 6 (27.4%) | 18 (16.2%) | 0.23 ⸸ |
| **Fibula** | 2 (9.1%) | 8 (7.2%) | p>0.40*** |
| **Pelvis** | 1 (4.5%) | 7 (6.3%) | p>0.40*** |
| **Sacroiliacs** | 2 (9.1%) | 13 (11.7%) | p>0.40*** |
| **Skull** | 0 (0%) | 1 (0.9%) | 0.05< p<0.10*** |
| **Humerus** | 1 (4.5%) | 5 (4.5%) | p>0.40*** |
| **Ulna** | 0 (0%) | 1 (0.9%) | 0.05< p<0.10*** |
| **Radius** | 1 (4.5%) | 5 (4.5%) | p>0.40*** |
| **Clavicle** | 1 (4.5%) | 3 (2.7%) | p>0.40*** |
| **Bones of the hand** | 0 (0%) | 2 (1.8%) | 0.10< p<0.15*** |
| **Foot bones** | 6 (27.4%) | 15 (13.5%) | 0.10< p<0.15*** |
| **Other** | 1 (4.5%) | 8 (7.2%) | p>0.40*** |

*SA: septic arthritis; OM: osteomyelitis; OA: osteoarthritis; SD: spondylodiscitis.

** One patient had no reported location *** Yates's chi-squared test ^ Chi-squared test ⸸Fisher's exact test
